# Epithelial specific splicing regulator proteins as emerging oncogenes in aggressive prostate cancer

**DOI:** 10.1038/s41388-023-02838-9

**Published:** 2023-09-26

**Authors:** Rahul Advani, Sara Luzzi, Emma Scott, Caroline Dalgliesh, Joachim Weischenfeldt, Jennifer Munkley, David J. Elliott

**Affiliations:** 1 Newcastle University Biosciences Institute (NUBI) and Newcastle University Cancer Centre, Newcastle University, Newcastle Upon Tyne, NE1 3BZ United Kingdom; 2 Biotech Research & Innovation Centre (BRIC), The Finsen Laboratory, Rigshospitalet, University of Copenhagen, Copenhagen, Denmark

**Keywords:** RNA splicing, Oncogenes

## Abstract

Prostate cancer progression is connected to the activity of conventional oncogenes and tumour suppressors and driven by circulating steroid hormones. A key issue has been how to identify and care for aggressively developing prostate tumours. Here we discuss how expression of the splicing regulators ESRP1 and ESRP2, and how their role as “masterminds” of epithelial splicing patterns, have been identified as markers of aggressively proliferating prostate primary tumours. We suggest that the origin of prostate cancer within epithelial cells, and the subsequent association of ESRP1 and ESRP2 expression with more aggressive disease progression, identify *ESRP1* and *ESRP2* as lineage survival oncogenes. To move this field on in the future it will be important to identify the gene expression targets controlled by ESRP1/2 that regulate prostate cancer proliferation. Potential future therapies could be designed to target ESRP1 and ESRP2 protein activity or their regulated splice isoforms in aggressive prostate tumours. Design of these therapies is potentially complicated by the risk of producing a more mesenchymal splicing environment that might promote tumour metastasis.

## Introduction

The prostate gland is located underneath the bladder in men and is the origin of the most frequent male-specific cancer [1, 2]. Prostate cancers can have variable disease progression: while some prostate tumours are relatively indolent and require monitoring alone, others can progress more aggressively and need to be treated with either radiotherapy or surgery. A key research priority is to identify the disease mechanisms that cause more aggressive cancers to develop, so that these can be detected and treated.

The molecular mechanisms driving prostate cancer have been intensively investigated, and involve hormone-driven signalling pathways, “classical” oncogenes including *ERG* and *MYC*, and tumour suppressors like *PTEN* [3, 4]. Since the 1940s it has been known that circulating levels of male steroid hormones called androgens (including testosterone and dihydroxy-testosterone) are critical drivers for prostate cancer progression [5]. Androgens exert their biological functions by interacting with a transcription factor called the androgen receptor (abbreviated AR). The AR protein is cytoplasmic in the absence of circulating androgens, but forms dimers in response to androgen exposure, and moves into the nucleus to control transcription of target genes important in cancer development. Approximately 50% of prostate cancers contain a genetic fusion between the androgen-responsive *TMPRSS2* gene and the *ERG* transcription factor gene. This genetic fusion places *ERG* (and therefore ERG-regulated downstream target genes that control prostate cancer cell proliferation, differentiation and apoptosis) under androgen control—thus converting ERG into a hormone-responsive oncogene [6, 7].

Many genes that are controlled by androgens have downstream effects on prostate cancer proliferation. For this reason, both locally advanced and metastatic prostate cancer are often treated with Androgen Deprivation Therapy (ADT) [3, 8], a treatment that reduces the production of androgens and promotes tumour regression. Currently used drugs used for ADT include luteinizing hormone releasing hormone (LHRH) antagonists that block the hypothalamic-pituitary gonadal axis of testosterone production. Other drugs include abiraterone, which interferes with enzymes required for androgen biosynthesis [9]; and bicalutamide (brand name Casodex) and enzalutamide (brand name Xtandi), which bind to the AR and blocks its ligand-dependent activity. Although ADT initially promotes prostate tumour shrinkage, cancers can relapse over time and become unresponsive to ADT—a condition described as castration-resistant prostate carcinoma (CRPC) [3].

Most human protein-coding genes are split into introns and exons, and are transcribed as precursor messenger RNAs (pre-mRNAs). Exons are spliced together to create mature mRNAs by a nuclear complex called the spliceosome [10]. Most human protein coding genes can be alternatively spliced to produce multiple different mRNAs. Alternative splicing plays a key role in prostate cancer progression, providing a possible angle of treatment, and this has been the topic of excellent recent reviews [11–13]. Much of the focus of alternative splicing research in prostate cancer has been on investigating alternatively spliced isoforms encoded by the androgen receptor gene (*AR*) itself. This is because alternative splicing can create *AR* splice variants that encode variant AR proteins that are constitutively active without androgens, and that become important later in disease progression as castrate resistant prostate cancer (CRPC) develops [11]. These include the splice variant ARv7 that includes a cryptic terminal exon, causing it to encode a variant androgen receptor containing a DNA binding domain but lacking a ligand binding domain. Expression of splice variants such as ARv7 contribute to androgen hormone-independent patterns of gene expression in patients with advanced prostate cancer and in response to ADT. How these AR splice variants are regulated has been the focus of intensive investigation [11]. Recent evidence indicates that regulation of epithelial splicing patterns is also important early in the disease progression of aggressive prostate cancer, and this is the topic of this review.

## Amplification of the *ESRP1* gene in early onset and highly aggressive prostate cancer

The most significant risk factor for a man to develop prostate cancer is age. The average age for prostate cancer diagnosis is 66 years, and prostate cancer is rarely found in men below the age of 40 years (https://www.cancer.org/cancer/prostate-cancer/about/key-statistics.html). Early onset aggressive prostate cancer (EOPC) develops in men below the age of 55 years, and this is likely to be the result of active oncogenic pathways driving tumour development. Identification and understanding of these oncogenic pathways is therefore of key importance. Genome analyses of prostate tumours have revealed amplification of the *ESRP1* gene (encoding the Epithelial Splicing Regulator Protein 1) to be associated with early onset aggressive prostate cancer [14]. Although the *ESRP1* gene is located on chromosome 8 close to the *MYC* oncogene (an important oncogene that is also frequently amplified in prostate cancer [15]), *ESRP1* amplification can be observed in prostate tumours independently of *MYC* alterations, indicating that it is not a bystander event [14]. Indeed, *MYC*-independent *ESRP1* amplification in early onset prostate cancer correlates with more advanced cancer histology and high mitotic activity of tumour cells, and is also predictive of a shorter time to disease recurrence [14]. Clonal analyses of tumour cells also show that *ESRP1* gene amplification occurs step-wise in the development of aggressive early onset prostate cancer, following genetic changes in tumour-suppressor genes such as *KLF5*, *BRCA1*, *RB1*, *PTEN* and *TP53* [14].

## ESRP1 and ESRP2 are “splicing masterminds” within epithelial cells

**Fig. 1:**
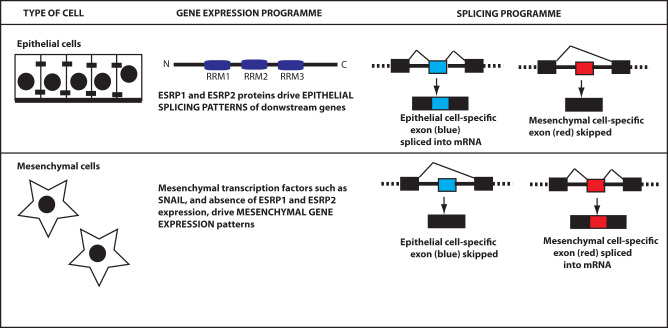
ESRP1 and ESRP2 are splicing masterminds of epithelial cells. Epithelial cells grow together in sheets of physically connected cells. Epithelial cells express high levels of ESRP1 and ESRP2 proteins that direct epithelial patterns of gene splicing. ESRP1 and ESRP2 contain three RNA Recognition Motif (RRM) domains that bind to target RNAs. Mesenchymal cells have a different morphology, tend to be mobile and have different patterns of gene expression from epithelial cells.

ESRP1 and its closely related paralog ESRP2 are known as “splicing masterminds” of epithelial cells [16]. Epithelial cells are tightly connected to adjacent cells and to basement membranes [17], and are anchored within monolayer sheets where they often function in secretion and boundary formation (Fig. 1) [18]. Epithelial cells typically grow in sheets within tissues, in close connection to other cells and often anchored to basement membranes. In contrast, mesenchymal cells usually exist as individual cells, and are able to move (Fig. 1). Human and mouse genomes each contain ESRP1 and ESRP2 proteins expressed by distinct paralog genes (with *ESRP1* on human chromosome 8, and *ESRP2* on human chromosome 16). Transcriptome analyses show that mouse organs and tissues containing a high proportion of epithelial cells also express high levels of both *ESRP1* and *ESRP2* (these high expressing tissues include the normal prostate) [19]. A major difference between *ESRP1* and *ESRP2* is at the level of overall expression. For example, the mouse *Esrp1* gene is expressed at higher levels than *Esrp2*, and genetic knockout of mouse *Esrp1* causes postnatal death due to cleft lip and palate [19]. In contrast, mice deleted for *Esrp2* are viable, while double knockout of *Esrp1* and *Esrp2* prevents many organs from properly forming [19], indicating ESRP1 and ESRP2 proteins can compensate for each other during normal development. Although ESRP1/ESRP2 proteins have mainly been investigated as splicing regulators within the nucleus, the human *ESRP1* gene can also produce an alternatively spliced transcript that encodes a cytoplasmic protein isoform that may regulate cytoplasmic aspects of epithelial function [20, 21].

Some key genes express alternative epithelial and mesenchymal mRNA splice isoforms that are specialised to function within their respective cell types. This means that the same gene will be made into a slightly different mRNA, often encoding a different protein isoform, depending on whether it is expressed in an epithelial or mesenchymal cell environment. ESRP1 and ESRP2 proteins regulate production of epithelial-specific alternative mRNA isoforms—explaining why they are known as “splicing masterminds” of epithelial cells. Human ESRP1 and ESRP2 proteins share an overall 60% amino acid identity. ESRP1 and ESRP2 proteins contain three domains called RRMs (RNA Recognition Motifs) that enable them to bind to pre-mRNA to control splicing patterns (Fig. 1) [22–24]. The RRM protein domains of ESRP1 and ESRP2 share higher levels of amino acid sequence conservation compared to their total protein sequences (RRM1: 88.5% identity; RRM2: 71% identity; RRM3: 80% identity). ESRP1 protein binds to a UG-rich RNA target sequence within introns and exons [25]. Binding of ESRP proteins to RNA within or immediately upstream of exons generally causes exon skipping, while ESRP binding within intron sequences downstream of regulated exons promotes splicing inclusion [18, 26]. Mesenchymal cells do not express ESRP1 or ESRP2, but do express specific transcription factors such as SNAIL (Fig. 1) that establish mesenchymal patterns of transcription.

## High expression of epithelial-specific splicing regulator proteins in primary prostate tumours correlates with a more aggressive disease trajectory

The study of EOPC provides a window into the development of aggressive prostate cancer, but EOPC only affects a small population of men. However, there is also data indicating that high levels of *ESRP1* and *ESRP2* gene expression more generally contribute to aggressive prostate primary tumour development and correlate with poorer patient prognosis. RNAseq data from general (i.e. not EOPC) primary prostate adenocarcinomas available in The Cancer Genome Atlas (TCGA) [27] show that high *ESRP1* (but not *ESRP2*) gene expression levels correlate with poorer patient prognosis [28–30]. Similar data have been obtained at the protein level by analysing tissue microarrays stained for ESRP1 and ESRP2. Immunohistochemistry of ~19,000 prostate tumours correlated high levels of ESRP1 and ESRP2 protein expression with more rapid biochemical tumour recurrence, higher Gleason scores, higher tumour stages and increased numbers of lymph node metastases [28, 29, 31].

## Androgens control ESRP1 and ESRP2 expression and their splicing targets in prostate cancer cells

Recent data have identified an important mechanism controlling ESRP1 and ESRP2 expression within prostate cancer cells that is linked to disease progression. Although androgens have been long known to control transcription patterns, the latest data show androgens also control alternative splicing [30, 32–34]. Two recent studies, one from our group (Munkley et al. [30]) and one by Shah et al. [32], identified a novel gene expression axis controlling epithelial splicing patterns in prostate cancer cells: androgen levels control expression of ESRP1 and ESRP2 proteins, which then control downstream epithelial splicing patterns [30, 32]. RNA-seq data from prostate tumours show that ADT reduces expression levels of both *ESRP1* and *ESRP2* genes [30, 32]. Furthermore, *ESRP2* expression can be directly induced within prostate cancer cell lines by exposure to androgens [30]. ESRP1 protein levels detected on tissue microarrays also directly correlate with pre-operative PSA levels, further linking androgen-driven gene expression with ESRP1 [14].

Importantly, many ESRP-regulated exons in prostate cancer cells have reciprocal splicing patterns when prostate cancer cells were stimulated with androgens versus treatment with a drug called Casodex that is used to antagonise the androgen receptor [30]. Expression levels of *ESRP1* and *ESRP2* genes also decrease in response to enzalutamide, a drug blocking AR function in prostate cancer cells [32]. As a consequence, treatment with enzalutamide also changed ESRP-regulated splicing patterns [32]. Treatment of cultured prostate cancer cells with Casodex and enzalutamide model the molecular changes that occur during ADT, and has established a clear link between ESRP1/ESRP2 and androgens.

The mechanisms through which ESRP1 and ESRP2 are controlled by androgen exposure have not been fully elucidated. The simplest model would be if *ESRP1* and *ESRP2* are direct transcriptional activated targets of the AR protein, and genomic ChIP (chromatin immunoprecipitation) identified AR binding sites within the vicinity of the *ESRP2* gene [30]. Analysis of ESRP1/ESRP2 protein staining in thousands of prostate tumours show that levels of ESRP1/ESRP2 proteins are highest in prostate cancers that are also positive for the *TMPRS22-ERG* genetic fusion [28]. Thus, ESRP1 and ESRP2 expression could also be indirectly activated via androgen-regulation of the ERG transcription factor in prostate tumours containing the *TMPRS22-ERG* genetic fusion. However, arguing against this indirect model, other immunohistochemical analyses have shown that higher levels of ESRP1 protein correlate with a shorter time to prostate cancer biochemical recurrence independent of ERG translocation status [14].

Each of the above bits of evidence indicate that increased expression levels of *ESRP1* and *ESRP2* in primary prostate tumours are associated with a more aggressive disease trajectory. This means that immunohistochemical analyses linking ESRP1 and ESRP2 expression with more aggressive disease progression might also be useful as prostate cancer biomarkers to apply in parallel to Gleason grading [28]. Such independent markers are needed since although Gleason grades are a good prognostic marker, the same tumours can be frequently assessed differently by different histopathologists [28].

## High levels of ESRP1 and ESRP2 in primary prostate tumours correlate with a more proliferative gene expression profile

An intriguing possibility is that the epithelial splicing patterns established by increased levels of ESRP proteins in response to circulating levels of androgens in prostate cancer patients could be identical to those splicing patterns established by *ESRP1* gene amplification in early onset prostate cancer. Thus, upregulation of *ESRP1/ESRP2* gene expression in aggressive tumours, by either gene amplification or in response to androgens could be different means to the same end, and drive the progression of aggressive prostate cancer in a similar way. This means that identifying the precise targets and functional consequences of the AR-ESRP gene expression axis could make a big contribution to understanding the biological basis of aggressive prostate tumour development.

**Fig. 2:**
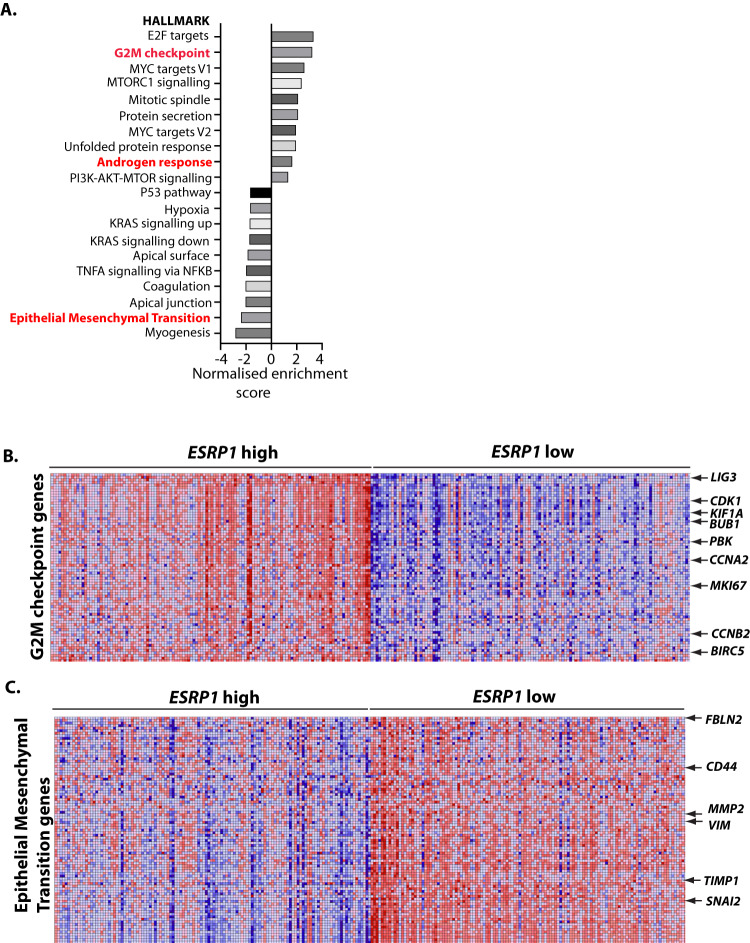
High levels of ESRP1 and ESRP2 in primary prostate tumours correlate with a more proliferative gene expression profile. **A** Analysis of the transcriptomes of 250 primary prostate tumours in the cancer genome atlas (TCGA) PRAD cohort identified enriched gene expression sets that correlate with *ESRP1* levels. The bar chart shows positively and negatively enriched sets of gene expression in primary prostate tumours stratified by ESRP1 gene expression levels (highest and lowest 25%). **B** Heat map showing higher expression of genes associated with the G2M transition in *ESRP1* high versus *ESRP1* low tumours. **C** Heat map showing reduced expression of EMT-associated genes in *ESRP1*-high tumours versus *ESRP1*-low tumours.

What could be the selective advantage for more aggressive prostate tumours to express higher levels of ESRP1 and ESRP2? Increased expression of ESRP1 and ESRP2 direct epithelial splicing patterns, and epithelial cells have been reported to replicate more rapidly than mesenchymal cells [35, 36]. Tissue microarray data show that high levels of ESRP1 correlate with an increased cellular proliferation rate [14]. Hence, by expressing higher levels of ESRP1 and ESRP2, more aggressive primary prostate tumours may maintain an epithelial gene expression environment to result in a growth advantage. Supporting this, Gene Set Enrichment Analysis (GSEA) of gene expression patterns in 250 primary prostate tumours with high levels of *ESRP1* in the TCGA PRAD cohort [27] revealed positive enrichment for gene sets associated with DNA replication and mitotic activity (Fig. 2A). Consistent with the above studies linking *ESRP1/ESRP2* gene expression with testosterone, the gene expression sets associated with high levels of *ESRP1* or *ESRP2* gene expression include the androgen response (Fig. 2A).

Distinct patterns of gene expression within tumours with elevated or reduced levels of *ESRP1* expression are illustrated by the heat maps in Fig. 2B, C. These heat maps show individual levels of gene expression activity. Tumours expressing elevated levels of *ESRP1* also have higher activity of genes associated with G2M checkpoints, compared to tumours expressing lower levels of *ESRP1* (high gene expression is indicated by red, and lower gene expression levels in blue in Fig. 2B, C). The genes with increased expression levels in *ESRP1* high tumours include *MKI67* (encoding the proliferation marker Mki67 [37]); the cell cycle regulator encoding genes *CDK1*, *CCNA2*, and *CCNB2;* and *BIRC5* that inhibits apoptosis.

The possession of epithelial and mesenchymal states are somewhat plastic, and are controlled by expression levels of ESRP1 and ESRP2 and the levels of mesenchymal transcription factors [38, 39]. Metastasis (the movement of cells from the primary tumour to other anatomic locations) involves a switch in cell morphology from epithelial to mesenchymal cells, called an Epithelial to Mesenchymal Transition or EMT. The biological changes that occur during metastasis mirror similar changes in cell properties that happen during normal development and wound healing (reviewed by [40]). ESRP1 and ESRP2 are key players in maintaining the epithelial gene expression environment. Experiments in human non-small cell lung cancer cells have shown that many (but not all) of the splice changes involved in EMT are directly controlled by changing concentrations of ESRP1 and ESRP2 [26].

Further meta-analysis of prostate cancer TCGA data show down-regulation of EMT pathways in primary prostate tumours expressing high levels of *ESRP1* (Fig. 2A). In contrast, lower levels of *ESRP1* correlate with patterns of gene expression associated with mesenchymal patterns of gene expression, including the mesenchymal transcription factors *VIM* and *SNAI2* (Fig. 2C). Interestingly, established metastases can de-differentiate to regain epithelial properties [39]. Hence re-expression of ESRP1 and ESRP2 proteins could in principle be part of a gene expression programme enabling successful metastatic growth once secondary tumours have seeded. Experimental analysis using cells and tissues from outside of the prostate show that *ESRP1* expression is transcriptionally controlled during metastasis. The mesenchymal transcription factor SNAIL binds within the *ESRP1* promoter to repress its transcription, and induce EMT of human mammary epithelial cells [41]. There are also changes in the chromatin architecture of the *ESRP1* gene following ectopic expression of the mesenchymal transcription factor Twist1 in mammary epithelial cells [42]. Inhibition of Twist1 promotes successful growth of mouse squamous cell carcinomas once metastases are established [36]. Such dynamic cell status switches between epithelial and mesenchymal cell gene expression programmes could be important within metastases, since epithelial cells proliferate more rapidly than mesenchymal cells, and mesenchymal transcription factors can inhibit genes important for cell growth [39]. Conversely the mesenchymal state is more compatible with increased cell mobility, drug efflux and immune evasion [39].

## ESRP1 and ESRP2 as lineage-survival oncogenes in early aggressive prostate cancer

**Fig. 3:**
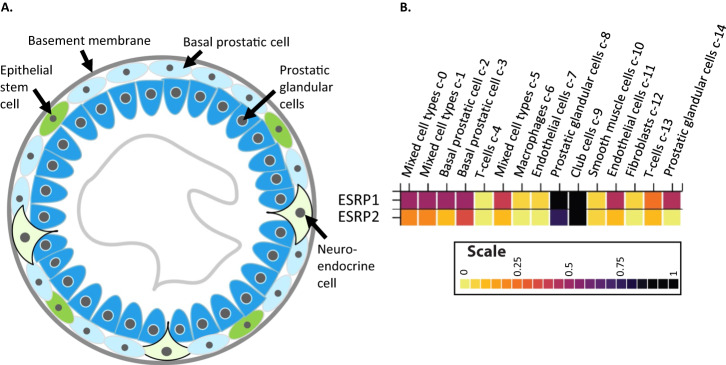
ESRP1 and ESRP2 as lineage survival oncogenes. **A** The cellular organisation of the prostate gland. **B** Cell type expression profiles of *ESRP1* and *ESRP2* from single cell RNAseq data downloaded from the Human Protein Atlas. Epithelial Cells (high levels of *ESRP1*/*2* expression): Luminal Secretory cells, Basal cells, Neuroendocrine cells, Club cells, and Hillock cells. Stromal cells (low levels of ESRP expression): Smooth muscle cells, T cells and fibroblasts.

Why might primary prostate tumours express high levels of ESRP1 and ESRP2 as a strategy to increase tumour growth? The answer to this question might be found in the normal cell biology of the prostate gland. The healthy prostate gland cell population is dominated by epithelial cells surrounding a central lumen (Fig. 3A). These epithelial cells include basal cells on the glandular periphery that also contain a stem cell population, and luminal cells which secrete fluid into the central lumen that nourishes sperm and helps their mobility and survival.

Prostate cancers usually initiate as a prostatic intraepithelial neoplasia, which then forms a localized prostate carcinoma. Most prostate tumours originate within the prostatic glandular cells and basal cells of the prostate gland, that have been identified from single RNAseq data to have high levels of ESRP1 gene expression (Fig. 3B) [3, 43]. Highly aggressive hormone resistant prostate cancers have overall gene expression profiles that resemble basal rather than luminal cell signatures, and prostate cancer cells can also trans-differentiate into neuroendocrine cells in response to androgen deprivation [44, 45]. However, whether prostate cancer cells derive from luminal, basal or trans-differentiated neuroendocrine cells, importantly an epithelial cell type of origin is still involved, and so this originating cell would be expected to have an epithelial splicing programme directed by ESRP expression (Fig. 3).

The fact that prostate tumours originate from epithelial cells that already express *ESRP1* and *ESRP2*, and then the *ESRP1* and *ESRP2* genes subsequently become important within a disease context, fits with the definition of a specific group of oncogenes called lineage-survival oncogenes [46]. By definition, lineage-survival oncogenes are important originally for the operation of healthy cell lineages but then become oncogenes during cancer initiation. Other known lineage-specific oncogenes include *SOX2* in lung and oesophageal squamous cell carcinomas, and *MITF* (microphthalmia-associated transcription factor) in melanoma [46, 47].

## Implications of ESRP1 and ESRP2 expression patterns for treating aggressive prostate tumours

Global RNAseq studies have identified multiple splicing targets for ESRP1 and ESRP2 in prostate cancer cells (Table 1) [30, 32]. Analysis of RNA expression data from prostate cancer patients RNA available in The Cancer Genome Atlas shows that expression of some of these ESRP-regulated splice isoforms correlates with a less favourable patient outcome [30, 32]. ESRP1 and ESRP2 both activate splicing of *NUMB* exon 3, and splicing of this exon in prostate tumours correlates with a reduced time until biochemical tumour recurrence in patients [30]. *NUMB* encodes a tumour suppressor protein that binds to MDM2 protein to prevent degradation of p53 and also inhibits Notch signalling [30, 48]. Other examples of ESRP-regulated splicing events that correlate with poorer patient prognosis include repression of exon 23 of the *MYO1B* gene [30]. The *MYO1B* gene encodes for an actin filament motor protein involved in intra-cellular vesicle transport. Exon skipping of *MYO1B* exon 23 correlates with a shorter time to prostate tumour biochemical recurrence [30].

**Table 1:** Some ESRP1/ESRP2 regulated splice events detected in prostate cancer cells.

GeneGene	Protein Function	ESRP regulation	Clinical relevance
*ADAM15*	Transmembrane glycoprotein involved in cell adhesion	*Activates inclusion of exon* 20	ESRP controls poorer prognosis isoform
*MINK1*	Serine/threonine kinase	*Activates inclusion of exon* 18	ESRP controls poorer prognosis isoform
*MYO1B*	Motor protein	*Represses exon* 23	ESRP controls poorer prognosis isoform
*NUMB*	Tumour suppressor	*Activates inclusion of exon* 6	*NUMB* exon 6 shows more skipping in primary prostate tumours
*MLPH*	Rab effector protein	*Represses exon* 9	ESRP controls poorer prognosis isoform
*MYH10*	Non-muscle actin dependent myosin	*Represses exon* 6	ESRP controls poorer prognosis isoform
*RPS24*	needed for cell proliferation	*Represses exon* 5	ESRP controls poorer prognosis isoform
*TUFT1*	adaptation to hypoxia, mesenchymal stem cell function, and neuronal differentiation	*Activates inclusion of exon* 2	ESRP controls poorer prognosis isoform
*DOCK7*	guanine nucleotide exchange factor	*Represses inclusion of exon* 23	*DOCK7* exon 23 shows more skipping in primary prostate tumours
*ARFGAP2*	ADP Ribosylation Factor GTPase Activating Protein 2	*Activates inclusion of exon* 8	*ARFGAP2* exon 8 shows more inclusion in primary prostate tumours
*ENAH*	cell motility and adhesion	*Activates inclusion of exon* 11*A*	*ENAH* exon 11 A shows more inclusion in primary prostate tumours
*FN1*	cell adhesion and maintenance of cell shape	*Represses exon* 25	
*FLNB*	Connects cell membrane to actin cytoskeleton	*Activates inclusion of exon* 30	Exon 30 included form in primary prostate tumours correlates with better prognosis
*MAP3K7*	controls transcription and apoptosis	*Activates inclusion of exon* 12	MAP3K7 genetic deletion leads to poor clinical prognosis

This selection of regulated splice events are taken from Munkley et al. 2019 [30] to illustrate how ESRP1/ESRP2 expression levels could control aspects of prostate cancer biology.

Since ESRP1 and ESRP2 are over-expressed in aggressive prostate tumours, could they also be targeted to reduce the growth of primary prostate tumours? Modulating expression of ESRP1 and ESRP2 in aggressive tumours could inhibit cell proliferation, but also have unintended side effects. The splicing patterns of *NUMB* exon 3, *MYO1B* exon 23 and other ESRP-target exons can be experimentally modulated either by depleting ESRP1 and ESRP2 protein levels in prostate cancer cells, or by using drugs like Casodex to interfere with AR signalling [30]. However, a complication of this possible approach is that any treatment that decreases activity of ESRP1 and ESRP2 may also dampen epithelial splicing patterns. This in turn could generate a prostate cancer cell that could metastasise more easily. Supporting this scenario in prostate cancer, ADT treatment has been linked to increased chance of metastasis in prostate cancer [49, 50]. This increased metastatic potential could be related to splice isoform production. For example, a splice isoform is produced from the *MAP3K7* gene (that encodes a mitogen activated protein kinase enzyme) that skips exon 12 in response to ESRP1/ESRP2 depletion in prostate cancer cells. This splice isoform, called *MAP3K7*Δexon12, is normally expressed in highly metastatic cancer cell lines [30]. The *FLNB* gene (encoding an actin binding protein) makes two different mRNA isoforms, one including exon 30 (made in epithelial cells) and one skipping exon 30 (made in mesenchymal cells). Increased skipping of *FLNB* exon 30 (an exon normally activated by the AR-ESRP axis in prostate cancer), is tightly linked to the development of metastases in breast cancer [51]. CRISPR-mediated deletion of *FLNB* exon 30 from the genome of breast cancer cells is sufficient to convert breast cancer cells from an epithelial to a mesenchymal phenotype [51].

## Changes in *ESRP1* and *ESRP2* expression are part of more global changes in the splicing environment during prostate cancer progression

Almost 68% of splicing regulator protein genes are differentially expressed over prostate cancer development, and there are also parallel changes in splicing patterns [52]. Hence expression changes in *ESRP1* and *ESRP2* in clinical prostate cancer disease progression, and downstream modulation of mRNA splicing patterns, most likely occur within the broader context of global changes taking place in the splicing environment [11, 53].

## Could ESRP1 and ESRP2 be lineage survival oncogenes in other kinds of tumour?

Could ESRP1 and ESRP2 also operate as lineage-survival oncogenes during the development of other tumours, many of which also develop from epithelial tissues? Supporting this, several studies indicate that ESRP1 overexpression plays a crucial role in various cancers where they also correlate with poorer survival statistics [24]. Higher levels of ESRP1 expression have been detected in breast cancer, and have been associated with poor prognosis in oestrogen receptor positive breast cancer cells [54]. *ESRP1* is controlled by the steroid hormone receptor ERα in breast cancer cells, and there are ERα binding sites within *ESRP1* and *ESRP2* promoters [55]. *ESRP1* is also controlled by the transcription factor E2F1 in breast cancer cells and sensitive to oxygen levels [56]. ESRP1 protein regulates splicing of the *CD44* cell surface receptor mRNA, that encodes a protein which interacts with tyrosine kinases in breast cancer cells [57]. The *CD44* gene contains group of variable exons which are repressed by ESRP1, to produce a splice variant called CD44s which positively correlates with co-expression of genes involved in cell proliferation [57]. Loss of ESRP1 expression leads to inclusion of these *CD44* variable exons, leading to production of variant CD44v protein isoform that is able to activate the PDGFRβ/Stat3 intracellular signalling pathway to promote the formation of a “cancer stem cell” population with mesenchymal properties [57]. Higher expression of *ESRP1* also results in poor survival in non-small-cell lung cancer [58] and in colorectal carcinoma [59]. ESRP1 is overexpressed in early stage colon cancer, and ESRP1 downregulation leads to colon cancer cell death [60].

## Conclusions

In summary, prostate cancer initiates within epithelial cell types. While androgen hormone signalling and classical oncogenes play a key role in prostate cancer development, recent data link the development of more aggressive primary prostate cancers with high levels of epithelial splicing regulator protein expression. ESRP1 and ESRP2 are encoded by genes that are important within the epithelial cells from which prostate tumours are derived, and then play an important role in subsequent tumour development. This gene expression pattern fits within the definition of a group of oncogenes called lineage-survival oncogenes. Future work will be needed to more precisely identify the genes controlled by ESRP1 and ESRP2 that are important in aggressive prostate cancer, how they result in tumour growth, and how expression of these genes are modulated in response to ADT and during tumour metastasis. Important questions for the future include whether the specific splice isoforms that cause aggressive prostate cancer properties can be identified and then therapeutically targeted.
